# A Perception on Genome-Wide Genetic Analysis of Metabolic Traits in Arab Populations

**DOI:** 10.3389/fendo.2019.00008

**Published:** 2019-01-28

**Authors:** Prashantha Hebbar, Jehad Ahmed Abubaker, Mohamed Abu-Farha, Jaakko Tuomilehto, Fahd Al-Mulla, Thangavel Alphonse Thanaraj

**Affiliations:** ^1^Genetics and Bioinformatics Unit, Dasman Diabetes Institute, Kuwait City, Kuwait; ^2^Doctoral Program in Population Health, Faculty of Medicine, University of Helsinki, Helsinki, Finland

**Keywords:** Arab population, type 2 diabetes, genome-wide association studies, risk loci, Kuwait, Euro-centric risk variants, genetics, epigenetics

## Abstract

Despite dedicated nation-wide efforts to raise awareness against the harmful effects of fast-food consumption and sedentary lifestyle, the Arab population continues to struggle with an increased risk for metabolic disorders. Unlike the European population, the Arab population lacks well-established genetic risk determinants for metabolic disorders, and the transferability of established risk loci to this population has not been satisfactorily demonstrated. The most recent findings have identified over 240 genetic risk loci (with ~400 independent association signals) for type 2 diabetes, but thus far only 25 risk loci (*ADAMTS9*, ALX4, *BCL11A, CDKAL1, CDKN2A/B*, COL8A1, *DUSP9, FTO, GCK, GNPDA2, HMG20A, HNF1A, HNF1B, HNF4A, IGF2BP2, JAZF1, KCNJ11, KCNQ1, MC4R, PPAR*γ*, SLC30A8, TCF7L2, TFAP2B, TP53INP1*, and *WFS1*) have been replicated in Arab populations. To our knowledge, large-scale population- or family-based association studies are non-existent in this region. Recently, we conducted genome-wide association studies on Arab individuals from Kuwait to delineate the genetic determinants for quantitative traits associated with anthropometry, lipid profile, insulin resistance, and blood pressure levels. Although these studies led to the identification of novel recessive variants, they failed to reproduce the established loci. However, they provided insights into the genetic architecture of the population, the applicability of genetic models based on recessive mode of inheritance, the presence of genetic signatures of inbreeding due to the practice of consanguinity, and the pleiotropic effects of rare disorders on complex metabolic disorders. This perspective presents analysis strategies and study designs for identifying genetic risk variants associated with diabetes and related traits in Arab populations.

## Background

Discovery of oil reserves in the Arabian Gulf since the 1930s increased overall wealth in these countries. Rapid socioeconomic transitions in Arab countries in the rich post-oil era marked changes in the nutritional patterns and food habits, including a shift from locally grown natural products to a Western diet and change from nomadic way to urbanized life. These resulted in an increasingly sedentary lifestyle and wide-spread obesity (1–3) and an increased prevalence of diabetes (4, 5), and metabolic syndrome (6) in the last few decades. Diabetes is sweeping through Middle East; as per the International Diabetes Federation Atlas 8th Edition for 2017, the age-adjusted comparative prevalence of diabetes (18–99 years) in the Middle East and North Africa region is 10.5%, which is the second highest after the North America and Caribbean region. Up to 30% of native adult hospital visitors in Kuwait are afflicted with T2DM (4). The prevalence among adults in the countries from the Peninsula (Kingdom of Saudi Arabia 31.6%, Oman 29%, Kuwait 25.4%, Bahrain 25.0%, and United Arab Emirates 25.0%) are significantly associated with high per capita GDP (gross domestic product) and energy consumption (7). T2DM results from a complex interplay of adverse lifestyle exposures and genetic predisposition. Despite the high prevalence of T2DM, the Arab population lacks convincingly determined T2DM genetic risk variants and reports that sufficiently demonstrate the replication of established risk variants.

### Identified T2DM Risk Loci in Arab Population Compared With Established Gene Loci

Since the advent of genome-wide association (GWA) studies, many T2DM risk loci have been globally identified, which concentrated mainly on the European and Asian populations. A recent study by Mahajan et al. (8), that combined data from 32 European-descent GWAS imputed to high-density reference panels, identified 243 genome-wide significant T2D-risk loci with 403 independent association signals. Therefore, it is important to understand the relevance and performance of these established loci in the Arab population and how often such novel risk loci are identified in this population. Published reports on T2DM genetics in the Arab population originate from Kuwait, Lebanon, Saudi Arabia, Qatar, UAE, Oman, and Tunisia (Table 1). Some of these studies illustrate certain overlaps with established T2DM risk loci, some illustrate the complete absence of any overlap (Table 1), and some identify novel risk loci. Approximately 25 established T2DM loci (*ADAMTS9*, ALX4, *BCL11A, CDKAL1, CDKN2A/B*, COL8A1, *DUSP9, FTO, GCK, GNPDA2, HMG20A, HNF1A, HNF1B, HNF4A, IGF2BP2, JAZF1, KCNJ11, KCNQ1, MC4R, PPAR*γ*, SLC30A8, TCF7L2, TFAP2B, TP53INP1, and WFS1*) and few established obesity loci (*ADIPOQ, FTO, RFX7, and USP37*) are observed to replicate in the Arab population (Table 1). The replicated T2DM risk loci fall into three categories (Table S1): (i) those that impact the T2DM risk through impaired β-cell function (*ALX4, BCL11A, CDKAL1, CDKN2A/B, GCK, HNF1A, HNF1B, HNF4A, IGF2BP2, JAZF1, KCNJ11, KCNQ1, SLC30A8, TCF7L2, WFS1*); (ii) those that act through modulating insulin action *(ADAMTS9, DUSP9, PPAR*γ)*;* and (iii) those that were primarily associated with BMI, obesity and adiposity but subsequently identified also as affecting T2DM risk through insulin action (*FTO, GNPDA2, MC4R, TFAP2B*).

**Table 1:** Studies on T2DM risk loci from the region and comparison with established T2DM loci.

**Study**	**Testing of established loci or general GWAS study; Study population**	**Sample size**	**Replicates known markers or identifies novel markers?**	**Conclusions**
**TARGETED GENOTYPING—REPLICATION STUDIES**				
Li-Gao et al. (9)	Tested 122 T2DM risk variants (from 84 loci) in Saudi Arabian population	659 T2DM cases and 919 controls	**Absence of any overlap:** None of the tested markers was replicating at *p*-value corrected for Bonferroni threshold (0.05/122). At *p* < 0.05, 11 were seen replicating	Failure to replicate any of the tested 122 risk variants was attributed to low sample size and also to study design—controls were not age-matched. The authors recommend large-scale GWAS
Osman et al. (10)	Tested established loci (BMI:87; WC:58; obesity with T2DM:145) in UAE Arab population	880 BMI cases; 455 WC cases; 464 T2DM cases and 415 contols	**Partial overlap and novel loci:** Replicates *FTO, USP37*, and *RFX7 (*for obesity*)* and *TCF7L2* and *MC4R (*for T2DM). Also reports novel associations *KCNK3* and *RARB* for T2DM	Could replicate very few established obesity and T2DM loci; the study could identify few novel associations for T2DM in Arabs
Cauchi et al. (11)	Tested 44 variants from 37 established loci for T2DM in North African Arabs (Morocco and Tunisia)	1,193 T2DM cases and 1,055 controls from Morocco. Associations were then assessed in 1,446 T2DM cases and 942 controls from Tunisia	**Partial overlap:** 13 of 37 established gene loci confirmed in Moroccans and Tunisians. *BCL11A, ADAMTS9, IGF2BP2, WFS1, CDKAL1, TP53INP1, CDKN2A/B, TCF7L2, KCNQ1, HNF1A, FTO, MC4R*, and *GCK*.	Concludes sharing of T2DM risk loci between Europeans and North African Arabs
O'Beirne et al. (12)	Tested 37 variants from 29 established T2DM gene loci, and an additional 27 tag SNPs in Qatari population	1,124 T2DM cases and 590 controls	**Partial overlap:** Only *TCF7L2* of the tested 29 loci was seen replicating in Qatari population	Concludes that the genetic risks for T2DM are likely different in Qataris compared to Europeans and Asians
Al-Daghri et al. (13)	Tested 28 established T2DM loci in Saudi Arabian Population	1,166 T2DM cases and 1,235 controls	**Partial overlap:** Replicates 9 of the 28 tested established T2DM loci of *TCF7L2, WFS1, JAZF1, SLC30A8, CDKN2A/B, KCNQ1, HMG20A, HNF4A*, and *DUSP9*	Concludes overlap in T2DM risk loci across ethnicities irrespective of prevalence
Tomei et al. (14)	Tested 23 established obesity-related loci in Qatari population	804 Qatari individuals	**Partial overlap:** Could identify only two (*TFAP2B* and *GNPDA2*) of the tested 23 obesity loci	Concludes a different genetic profile associated with obesity in the Qatari population compared to Western populations
Almawi et al. (15)	Tested 19 SNPs in/near 15 established T2DM loci in Lebanese Levant population	995 T2DM cases and 1,076 controls	**Partial overlap:** 4 (*COL8A1, KCNQ1, ALX4, HNF1B*) of the 15 established loci were seen associated with T2DM in Levants. The authors have shown in their previous works replicability of IGF2BP2, CDKAL1, TCF7L2.	Concludes that insufficient power as the reason for the inability to detect all the tested loci in Levants
Al-Sinani et al. (16)	Tested 10 variants from 9 established T2DM loci—*KCNJ11, TCF7L2, CDKAL1, CDKN2A/B, FTO, IGF2BP2, SLC30A8, CAPN10, HHEX* in Omani Arabs	992 T2DM cases and 294 controls	**Partial overlap:** Only four of the 9 tested loci could be replicated—*KCNJ11, TCF7L2, CDKAL1, CDKN2A/B*	Suggests large-scale studies, other than case-control design, to detect rare variants that might explain the missing heritability
Mtiraoui et al. (17)	Tested 7 established T2DM loci - *ENNP1, IGF2BP2, KCNJ11, MLXIPL, PPARγ, SLC30A8*, and *TCF7L2* in Levant Arabs from Lebanese and North African Arabs from Tunisia	Lebanese: 751 T2DM cases and 918 controls Tunisia: 1,470 T2DM cases and 838 controls	**Partial overlap with established markers and differences in overlap between the two Arab popualtions:** *TCF7L2* was replicating in both; *IGF2BP2* and *PPARγ* were replicating in Lebanese and not in Tunisia; *KCNJ11*, and *SLC30A8* were replicating in Tunisia but not in Lebanese. Neither *ENNP1* nor *MLXIPL* was seen replicating in Lebanese or Tunisians.	Concludes differences in replicability of T2DM loci between Lebanese and Tunisians as well as between Europe and these two Arab groups.
El Hajj Chehadeh et al. (18)	Investigated the association between the *MTHFR* SNPs (C677T and A1298C) and T2DM in Emirati Arabs	169 T2DM cases and 209 controls	**Absence of any overlap:** *MTHFR* gene polymorphisms are not related to T2DM in the Emirati population	Could not establish MTHFR variants as risk variants for T2DM in Emirati population
Al Safar et al. (19)	Tested the T2DM risk loci—*TCF7L2 (*rs10885409*)* and *PPARγ2 (*rs1801282*)* in Arab Emirati population	272 T2DM cases and 216 controls	**Partial overlap:** Confirms the association of the *TCF7L2* variant but not *PPARγ2* as a T2DM loci in UAE Arabs	Confirms one of the two established markers as T2DM loci; and the other established marker as NOT a T2DM loci
Khan et al. (20)	Tested the established obesity loci—*FTO* (rs9939609) and *VDR* (rs1544410), in UAE population	201 obese, 115 overweight, and 98 normal subjects	**Partial overlap:** Replicates the *FTO* marker as adult obesity risk loci in UAE. *VDR* could not be replicated	No significant association is seen in UAE population for the established marker from *VDR*
**POPULATION-BASED GWA AND FAMILY-BASED STUDIES**				
Ghassibe-Sabbagh et al. (21)	Performed GWAS (on genotyped and imputed markers) in Lebanese population	3,286 Lebanese participants	**Partial overlap and Novel loci:** Only two established loci (*CDKAL1* and *TCF7L2*) surfaced	Concludes that the replication of established markers in Lebanese population is less than expected
Al Safar et al. (22)	Performed family-based GWAS for T2DM in UAE Arabs	Discovery cohort: *N* = 178 (66 cases, 112 controls); Replication cohort: *N* = 315 (116 cases and 199 controls)	**Novel loci:** Identified novel associations (*KCTD8, PRKD1, GABRA2, GABRA4*, and *GABRB1*)	Did not identify any of the established gene loci
Zadjali et al. (23)	Family-based study (“Oman Family Study”) to investigate the association of SNPs (rs17300539 and rs266729) from adiponectin gene *ADIPOQ* with obesity traits in Oman	328 Arabs in one large extended family from Oman	Showed family-based evidence for association of one (rs266729) of the two tested SNPs from *ADIPOQ* defining obesity in Arab population	Concludes that *ADIPOQ* as obesity loci in Arabs from Oman
Our own studies (24–26)	Performed GWAS for quantitative metabolic traits on Arabs from Kuwait	Discovery cohort: 1,353; Replication cohort: 1,176 from Kuwait	Identified novel associations for metabolic traits	Only three established markers from *CETP* and *STARD3* are seen at borderline *p*-values

Studies illustrating a replication of established risk loci in Arab population are generally based on targeted genotyping, whereas those failing to observe the established loci are GWA-based. GWA studies for quantitative traits in the Arab population in Kuwait were unsuccessful to demonstrate established loci at genome-wide significance but instead led to identification of novel risk loci (Table 2); these identified novel loci are often reported in literature as associated with biological processes relating to the T2D traits (Table S2). Moreover, even at nominal *p*-values, very few established markers are identified in the data sets in the Kuwaiti population (Table 2—Items V–VI). The exemplary susceptibility gene loci namely, *PPAR*γ*, KCNJ11, TCF7L2, SLC30A, ABCC8, HHEX, CDKN2A, IGF2BP2, CDKAL1*, and *FTO*, known to be well-replicated in other ethnic population groups were not identified in our studies on the Kuwaiti population. Similarly, only two established T2DM risk gene loci namely (*CDKAL1* and *TCF7L2*) were identified using an imputed data set of 5,000,000 single nucleotide polymorphisms (SNP) in the Lebanese population, which has a higher affinity to European populations compared with other Middle Eastern populations (21). It was also observed that the established markers do not necessarily replicate among inter-Arabic population groups. For example, Mtiraoui et al. (17) (Table 1) illustrated differences between the Levant Arabs (from Lebanon) and north African Arabs (from Tunisia) by demonstrating the association of *TCF7L2* in both groups, *IGF2BP2* and *PPAR*γ exclusively in the Lebanese group, and *KCNJ11* and *SLC30A8* exclusively in the Tunisian group.

**Table 2:** Risk variants identified in our previous studies on Arab individuals from Kuwait.

**Metabolic traits**	**Gene loci /variant**	**Model; *p*-value; Beta value for associations**	**OMIM annotation for the gene (where available)**
**I. OBESITY TRAITS (**25)			
Waist Circumference (WC)	*TCN2*/rs9606756	Additive; 1.46E-07 (9.46E-08 upon correction for medication); 4.815	Transcobalamin II deficiency (AR) (PMIM: 275350)
**II. BLOOD PRESSURE TRAITS (**27)			
Systolic Blood Pressure (SBP)	*MC3R*/rs3827103 [Val81Ile]	Additive; Sequencing the genes. 0.01; 4.9	Mycobacterium tuberculosis, protection against; (PMIM: 607948); Obesity, severe, susceptibility to, BMIQ9; (PMIM: 602025)
**III. METABOLIC TRAITS (**26)			
Glycated hemoglobin (HbA1c)	*ZNF106* (W > R)/rs12440118	Recessive; 7.07E-08; 2.006	
Fasting Plasma Glucose (FPG)	*OTX2-AS1*/rs7144734	Recessive; 2.82E-07; 1.465	OTX2 with Microphthalmia, syndromic 5 (AD) (PMIM: 610125); Pituitary hormone deficiency, combined, 6 (AD) (PMIM: 613986); Retinal dystrophy, early-onset, with or without pituitary dysfunction (AD) (PMIM: 610125). AS1 with susceptibility to Asthma (PMIM: 607277)
Triglyceride (TG)	*PLGRKT*/rs17501809	Recessive; 1.04E-07; 1.807	
Triglyceride (TG)	*LOC105376072*/ rs11143005	Recessive; 4.03E-07; 0.419	
Triglyceride (TG)	*IGF1*/rs10860880	Recessive; 2.07E-07; 1.596	Growth retardation with deafness and mental retardation due to IGF1 deficiency (AR) (PMIM: 608747). Decreased IGF-1 secretion occurs in the majority of the thalassemia patients particularly those with growth and pubertal delay (28). Thalassemia is recessively inherited
Triglyceride (TG)	[*THSD4, NR2E3*]/ rs900543	Recessive; 1.27E-07; 1.625	NR2E3 with Enhanced S-cone syndrome (AR) (PMIM: 268100); Retinitis pigmentosa 37 (AD,AR) (PMIM: 611131)
**IV. LIPID TRAITS (**24)			
Triglyceride/Fasting Plasma Glucose/Glycated hemoglobin—TG/FPG/HbA1c	*RPS6KA1*/rs1002487	Recessive; 7.17E-11; 6.517/1.64E-08; 8.315 (for FPG)	
Triglyceride (TG)	*LAD1*/rs11805972	Recessive; 2.16E-17; 8.485	Leukocyte adhesion deficiency (AR) (PMIM: 116920)
Triglyceride (TG)	*OR5V1*/rs7761746	Recessive; 1.31E-09; 6.006	
Triglyceride (TG)	[*CTTNBP2, LSM8*]/rs39745	Recessive; 1.57E-08; 5.643	
Triglyceride (TG)	*PGAP3*/rs2934952	Recessive; 1.16E-09; 6.086	Hyperphosphatasia with mental retardation syndrome 4 (AR) (PMIM: 615716)
Triglyceride (TG)	[*RP11-191L9,CERK*]/rs9626773	Recessive; 7.47E-15/7.776	
Triglyceride (TG)	*ST6GALNAC5*/rs10873925	Recessive; 4.11E-08/0.633	
Triglyceride (TG)	*SPP2_ARL4C*/rs4663379	Recessive; 8.38E-09/1.841	
Triglyceride (TG)	*NPY1R*/rs10033119	Recessive; 8.79E-09/2.698	
Triglyceride (TG)	*LINC00911_FLRT2*/rs17709449	Recessive; 5.12E-08/1.173	
Triglyceride (TG)	*CDK12-NEUROD2*/ rs11654954	Recessive; 2.18E-08, 0.9881	
Triglyceride (TG)	*STARD3*/rs9972882	Recessive; 1.81E-08, 0.7284	
**VI. ESTABLISHED MARKERS (AT SUGGESTIVE *p*-VALUES IN GWAS CATALOG) APPEARING IN KUWAITI DATA SET ALSO AT NOMINAL *p* -VALUES FOR ASSOCIATION**			
Triglyceride (TG)	rs9326246/*BUD13*	Additive; 5.19E-06; 0.24 (KWT) ≤ 1.27E-229; 0.22 [European population (29)]	
High-Density Lipoprotein (HDL)	rs3764261/*CETP*	Additive; 1.10E-05 (KWT)—reached 4.64E-08 under joint analysis. 1E-769 [European, East Asian, South Asian and African ancestry (30)]
High-Density Lipoprotein (HDL)	rs1864163/*CETP*	Additive; 4.64E-06 (KWT)—reached 1.15E-08 under joint analysis. 7E-39 [FUSION, SardINIA, Diabetes Genetics Initiative studies; also, in East Asian, and European populations (30, 31)]	
High-Density Lipoprotein (HDL)	rs1800775/CETP	Additive; 4.99E-06 (KWT)—reached 5.51E-08 under joint analysis. 4E-93 [Europeans and Filipinos (32)]	
Triglyceride (TG)	rs9972882/STARD3	Additive; 4.07E-07 (KWT) An LD marker rs1877031/ STARD3 is an established marker in East Asians for the related trait of HDL at 1E-21 [East Asians (30)]	
**VI. ESTABLISHED MARKERS (AT GENOME-WIDE SIGNIFICANCE IN GWAS CATALOG) APPEARING IN KUWAITI DATA SET AT NOMINAL *p*-VALUES FOR ASSOCIATION**			
Triglyceride (TG)	rs900543/[*THSD4,NR2E3*]	Recessive; 2.26E-07; 1.625 (KWT). 9.40E-05; 0.036 [Europeans (33)] Fasting insulin	
Triglyceride (TG)	rs11143005/*LOC105376072*	Recessive; 3.218E-07; 0.420 (KWT). 4.47E-05; 0.11 [Europeans (34)] 2 h fasting glucose	
Triglyceride (TG)	rs17569297/[*LOC105369738, LOC105369739*]	Recessive; 6.963E-06; 0.773 (KWT) 1.51E-06; NA [Europeans (35)] HDL	
Total Cholesterol (TC)	rs10935794/[*RPL32P9, LINC01213*]	Additive; 3.65E-06; 0.2037 (KWT) 9.80E-05; NA [Europeans (36)] Serum ratio of Arabinose fructose	

The partial overlap of established markers and differential replication of established markers in inter-Arabic populations along with the identification of novel loci are also observed in case of other complex disorders, such as rheumatoid arthritis, myocardial infarction/coronary artery disease, prostate cancer, and breast cancer (37–40). It is highly interesting to perform genetic association studies in ethnic populations such as Arabs, not only to re-confirm findings from other ethnic groups but since they may also lead to identification of novel T2DM risk loci.

## Possible Causes for Observed Differences in the Risk Loci Profiles

### Study Cohorts

The above-mentioned observations in Arab populations are associated with the size of study cohorts used in the studies, which is considerably lower than those used conventionally in global GWA studies (Table 1). This deficit affects the strength of the studies and leads to issues such as non-consideration of markers that have become rare in Arab populations. Other shortcomings of the study designs are inconsistencies (such as in age) between case and control cohorts and the failure to include in the set of markers tested for replication those that are in LD with established markers.

### Differences in Phenotype Profiles Between Arab and Global Populations

While most of the well-characterized T2DM genes appear to be associated with β-cell dysfunction, diabetes observed in the Arab population is supposedly associated with obesity. This is underlined by the following observations in the Arab population: the prevalence of obesity in T2DM patients is high (41), Arab studies often find obesity-related genetic loci (e.g., *ADIPOQ* gene) to contribute to the genetic risk of T2DM in Arab populations (10, 23, 42, 43), and established T2DM-related SNPs are seen associated with obesity phenotypes in Arabs (44).

### High Rate of Consanguinity and Prevalence of Rare, Mendelian, and Familial Disorders in Arab Populations

Marriages in Arab populations are traditionally often consanguineous (45, 46). An increased risk of T2DM has been observed among the offspring of such consanguineous marriages in Saudi Arabia and Qatar (47, 48). The familial clustering of T2DM has been reported in Arab populations from Morocco (49), Tunisia (50), Oman (51), and Qatar (52). Additionally, Arab populations exhibit many rare, Mendelian, and familial genetic disorders. Blair et al. (53) identified thousands of associations between Mendelian and complex diseases in the medical records of over 110 million patients from USA and revealed a non-degenerate, phenotypic code that links a complex disorder to a unique collection of Mendelian loci. Using GWA studies, they further demonstrated that common variants associated with complex diseases are enriched in the genes indicated by this “Mendelian code.” Thalassemia, cystic fibrosis, Huntington's disease, and Friedreich's ataxia are examples of rare disorders that increase patient's pre-disposition to diabetes (54–58). Examples of T2DM risk genetic loci, which are also associated with rare recessive disorders, are *LIPC* (Hepatic lipase deficiency), *PDX1* (Pancreatic agedness 1), *ENPP1* (Hypophosphatemic rickets, also associated with obesity), *WFS1* (Wolfram syndrome 1), and *SLC2A2* (Fanconi-Bickel syndrome). Table 2 summarizes OMIM disease annotations, from the OMIM (Online Mendelian Inheritance in Man) catalog of human genes and genetic disorders (59), for risk loci that were identified for metabolic traits in Kuwaiti Arab populations. The genetic locus *OTX2-AS1* associated with hypogonadism is particularly interesting as literature reports have provided hints regarding the connection between hypogonadism and T2DM in Arab consanguineous families (60, 61). Al Hayek et al. reported that 36.5% men with T2DM from Jordan had low serum testosterone levels; 17% of such T2DM patients with low serum testosterone levels had primary hypogonadism, whereas the remaining had secondary hypogonadism (62). A study based on Saudi Arabian population revealed a significantly higher positive family history of schizophrenia in patients with first or second cousin parents (63). An increased prevalence of T2DM has been observed in patients with schizophrenia (SCZ); the co-occurrence of SCZ and T2DM may partly be driven by shared genetic factors (64) such as cell adhesion molecules (65, 66). Furthermore, the QDiabetes-2018 model (used within the UK National Health Service) (67) now includes schizophrenia, learning disabilities, use of atypical antipsychotics, treated hypertension, and polycystic ovary syndromes as additional risk factors.

## Analysis Strategies for GWA Studies in Arab Populations

**Figure 1:**
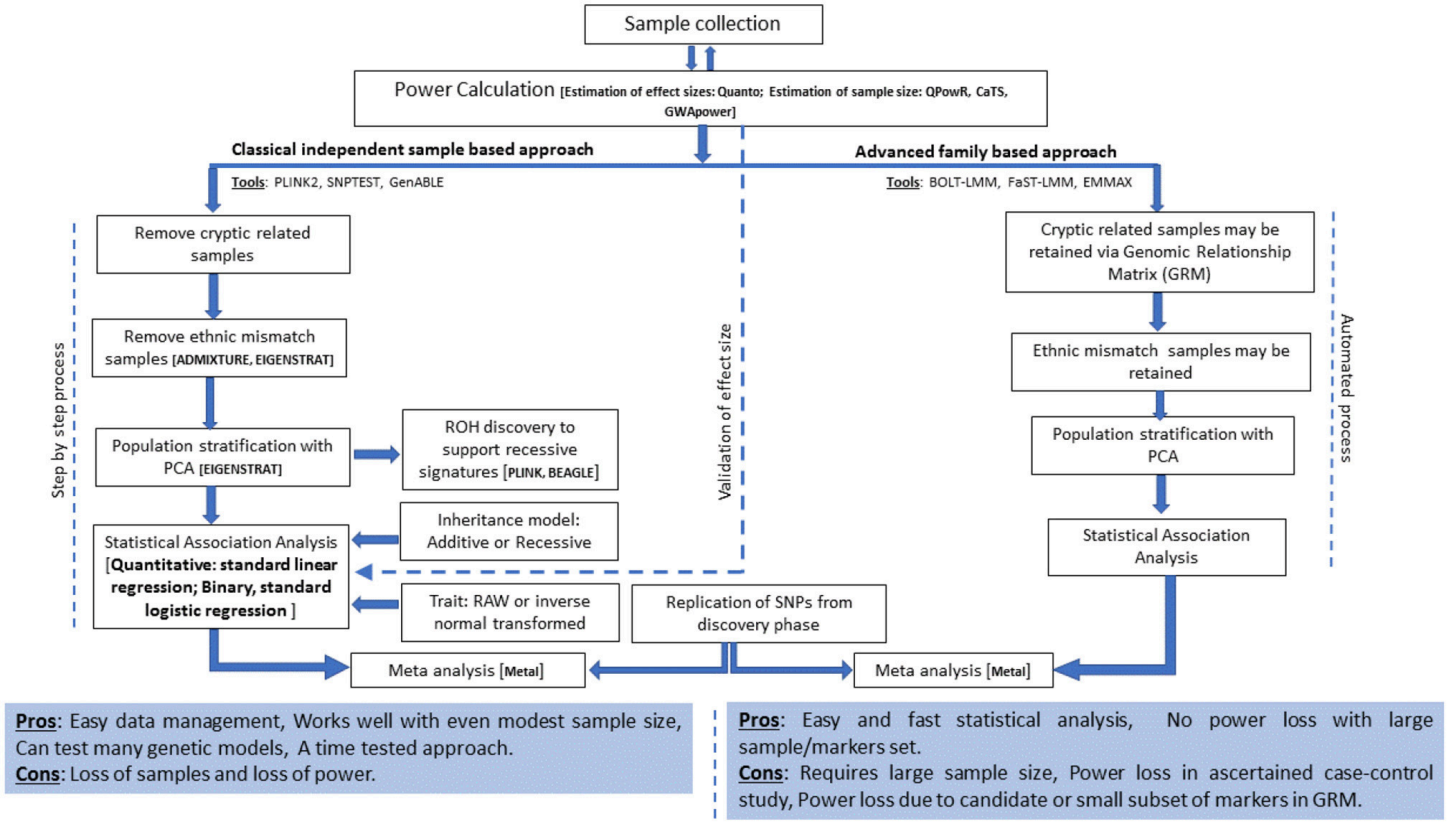
Current analysis strategies and suggested extensions for GWA studies in Arab populations.

The current analysis strategies for GWA studies in Arab populations and suggested extensions are presented in Figure 1 and are discussed below.

### Participant Recruitment and Sample Size

The sample size has a linear relationship with the number of identified risk loci; a plateau has not been observed for any trait to date (68). Recruiting the required large number of participants from the intended sample population continues to be challenging in Arab countries (69, 70). Clinical research in Arab countries experiences a lack of public outreach capabilities and coordination between research institutes and hospitals or Ministries of Health. This challenge can be circumvented by understanding public perception and attitude toward medical research and by seeking out means to increase public trust and awareness of clinical research in the Arab population.

The optimum sample size is determined by various factors including homogeneity seen in the population, prevalence of the disorder, variance in the trait measurements, genetic models used in the association tests, number of markers tested in the study, allele frequencies of the risk variants, effect sizes, genome control inflation rates, desired Type I error rates, type of study design (quantitative trait association, case-control studies with unrelated individuals or family-based trios, or sibling case-control designs) (71, 72). A number of tools are available to calculate the optimum sample size; some of the tools often used include Genetic Association Study (GAS) Power Calculator for one-stage genetic association studies http://csg.sph.umich.edu/abecasis/cats/gas_power_calculator/; (73), CaTS Power calculator http://csg.sph.umich.edu//abecasis/CaTS/index.html; (74) for two stage genome wide association studies; Quanto for various study designs (including the matched case-control, case-sibling, case-parent, and case-only designs) http://biostats.usc.edu/Quanto.html; (75), and QpowR for two-stage study design with unrelated individuals https://msu.edu/~steibelj/JP_files/QpowR.html.

### Differences in the Extent of Inbreeding Among Subgroups in the Arab Population

The rate of consanguinity and the extent of resultant inbreeding differ among Arab countries as well within a single country. For example, among the three genetic substructures of Kuwaiti Arab population (76–79), the subgroups of Saudi Arabian tribe ancestry and Persian ancestry exhibit high inbreeding coefficients (0.04226 and 0.025742, respectively) indicating endogamy, whereas the nomadic Bedouin subgroup exhibits lower inbreeding coefficient (0.00274), indicating heterogamy (76). Similar observations have been made with population substructures in Qatar (80). The genetic heterogeneity between the subgroups warrants subgroup-specific genetic association analysis using large sample sizes, particularly for those with higher rates of inbreeding.

### Comorbid Conditions as Confounders

Diabetes is often comorbid with other complex chronic disorders (81); Most prevalent comorbid disorders, that occur in “concordant” form wherein both disorders represent parts of the overall identical pathophysiological risk profile, include hypertension, coronary artery disease and peripheral vascular disease (82). An example for comorbid disorders being influenced by unique environmental factors is an increased risk for diabetes in patients with learning disabilities, physical or sensory disabilities, and mental health problems. The co-occurrence of schizophrenia and diabetes may partly be driven by shared genetic factors (64). Comorbidities can also be consequences of hyperglycemia. Tests for genetic associations need to consider comorbid conditions. For advancements in metabolic disorder research in the Arabian Peninsula, the identification of overlaps between metabolic disorders and rare genetic as well as common disorders from existing literature and the formation of a catalog of causal factors/markers for overlapping traits are immediately required. A careful and extensive phenotyping in relation to rare and less frequent disorders must be prioritized during sampling. These variables can be further used as covariates in association tests.

### Genetic Models Based on Recessive Mode of Inheritance for Association Tests

Consanguinity in successive generations cumulatively increases inbreeding levels, recessive alleles, and the proportion of homozygous gene regions in Arab populations (76, 83). An overwhelming proportion (63%) of the disorders documented in the Catalog for Transmission Genetics in Arabs (CTGA) (84) follows a recessive mode of inheritance. Therefore, it is not surprising to observe that the risk variants reported in our studies appeared when the genetic model based on a recessive mode of inheritance was used (Table 2), and majority of the risk variants were observed to be harbored in the runs of homozygosity (ROH) segments (24). However, when established markers appeared in the Kuwaiti population data sets, it was invariably when the association tests used additive models. It is obvious that genetic pre-disposition of T2DM is not a recessive disorder, although in certain populations homozygosity of susceptibility alleles can further increase the population prevalence of the disease. It is recommended that population genetics in the Arab region use recessive models in addition to additive models, especially when large number of homozygous segments and/or of recessive risk genotypes are observed in sufficiently large number of individuals in the study cohort.

### Estimation of Acceptable Effect Size

Large sample sizes enable the identification of causal variants with small effect size in GWA studies. Although the estimation of sample sizes for a 2-stage design (discovery phase followed by replication) is generally strictly followed in GWA studies, the estimation of a true effect size explainable by sample size is not strictly followed in several studies; the proper selection of associated variants in the discovery phase is not usually difficult, mainly because the use of additive models (wherein both heterozygote + rare homozygous genotypes are tested against reference homozygous genotypes with additive effect) does not unusually inflate the effect size values for common markers. However, in case of the recessive model, variants that show ≥5% frequency (i.e., associated with few rare homozygous genotypes) can show unusually large effect sizes. Therefore, an estimation of the acceptable effect size for a desired percentage of variance in a given trait (mean ± SD) and sample size at 80% power is pivotal for restricting SNP associations from undesirable high effect size and inappropriate *p*-values resulting from recessive effect.

### Joint Analysis—Combining Results From Discovery and Replication Phases

GWA studies follow a 2-stage design irrespective of addressing quantitative or binary trait association. A strategy involving the joint analysis of data from both stages, which is currently increasingly used, has been advocated to result in an increased capability of detecting genetic associations (74). Such a meta-analysis increases the capacity for detecting weak genotypic effects. An example of the resultant increased power is observed in the case of three established markers from *CETP* (rs3764261, rs1864163, and rs1800775), which do not attain genome-wide significance in discovery cohort of our studies but in joint analysis (Table 2—Item V).

### Heteroscedasticity and Trait Transformation

Quantitative traits (associated with complex disorders) used in association studies often violate the assumption of normality. Methods commonly used to handle non-normal traits include natural logarithm and rank-based inverse-normal transformations (85). Although the merits of such transformations are questionable (86), their use has been increasing. Elaborate assessments regarding the extent to which such transformations mask the true phenotype variability are lacking in literature. Owing to high inbreeding and prevalence of autosomal recessive disorders in the Arab population (87), the segregation of rare homozygous alleles may exert relatively larger effects on quantitative trait variations. Hence, earmarking any outlying data dispersion as heteroscedastic may conceptually be incorrect. Furthermore, performing trait transformations may adversely mask the actual effects of such loci on the variation of quantitative traits. Thus, to avoid any false positive or negative associations, it is advised to perform association tests, using appropriate genetic models, with both the raw and transformed traits, and to simultaneously adjust the models for disorders (rare and common) that overlap with the metabolic syndrome.

### Relatedness and Loss of Samples

The imprecise modeling of genetic relatedness and population stratification among study subjects results in a substantial inflation of test statistics and spurious associations. Moreover, randomized sample sets in the Arab population exhibit rich relatedness due to the prevalent practices of polygamy and consanguinity. Therefore, detailed quality control procedures for relatedness and admixture must be performed in case of Arab studies. Relevantly, our studies are performed using the following steps: (i) assessment of relatedness among participants to the extent of third-degree relatives and removal of one sample per pair of related participants, (ii) performing ancestry estimations using ADMIXTURE (88) and removal of samples with abnormal deviations to the extent of component ancestry elements that have been previously established for the three Kuwaiti population subgroups (76), and (iii) delineation of principal components using EIGENSTRAT (89) and removal of outlying samples. These exhaustive steps, aimed at reducing false positive findings, lead to huge loss of samples. Thus, although the use of unrelated individuals in GWA studies is a norm, the use of recent sophisticated algorithms [such as BLOT-LMM 90 FaST-LMM 91, EMMAX 92], which account for kinship structures and ancestries within a sample set, may offer larger power to the study by retaining more samples.

### Quantitative Trait Association Studies Using “All” Diabetic Cohort

Quantitative trait association studies are usually conducted in population-based cohorts comprising both people with diabetes and diabetes-free individuals at the time of participant recruitment. Association tests are usually adjusted for obesity, diabetes, and medication regimen. However, it is important to extend the studies to cohorts comprising entirely of diabetic or prediabetic participants so that the prospects for use of identified genetic determinants in diabetes care and treatment become promising (93).

### Case for Whole-Genome Homozygosity Association (WGHA) Methodologies

High inbreeding within the Arab population renders it a promising repository for providing a large scope for discovery of ROH and segments of identity by descent (IBD). ROH indicates an ancient shared common ancestry, whereas IBD indicates a recent ancestry. Both traits are reportedly effective in delineating population demography and recessive components of Mendelian and complex phenotypes (76). The risk loci identified in our studies often overlay ROH regions, some of which are “novel.” Currently, a promising new concept of “whole-genome homozygosity association (WGHA) methodology” in identifying genetic susceptibility loci harboring recessive variants (94) is being developed. Exemplary works include the identification of “risk ROH” for schizophrenia (95) and adult height (96). Tools such as LOHAS (97), which use either whole-genome sequence or genotype data in cohorts of either related or unrelated individuals, are now available for performing WGH association tests under the study designs for both case–control and quantitative trait association tests.

### Case for Family-Based Genetic Association Studies in Arab Population

The large number of T2DM genetic loci identified to date using unrelated people explains only a relatively small proportion of observed heritability (familial clustering) of T2DM (8, 98). Possible explanations for “missing heritability” may originate from the role of rare variants, copy number variants, indels and more complex rearrangements, gene-environment interactions and epigenetics (98–102). Family-based designs allow the segregation of rare variants in a pedigree; multiple copies of such rare variants facilitate the detection of their effects. Family-based studies require a fewer number of samples than population-based studies and offer advantages in terms of quality control, robustness to population stratification, and uniformity in exposure to environmental factors or lineage-specific diseases. They offer the potential to combine linkage and association data. Arab population, which is largely consanguineous, offers a large potential for family-based designs as the population can show familial gene clustering for diabetes and metabolic traits. However, except for few studies, such as the “Oman Family Study” (103–106) and the study on an extended family from the UAE by Al Safar et al. (22), no notable familial study for diabetes risk loci has been reported on the Arab population. Both the abovementioned studies confirmed well-established gene loci, but failed to identify any novel “rare” variants. Considerable attention needs to be paid to appropriate study designs as family data continue to provide important information in the search for trait loci (107). It is ideal if the recruitment of large-pedigrees/extended families, particularly those containing several sub-families suitable for both parent-offspring design or for sibling design, with high inbreeding and roots traceable up to at least six generations with deduced consanguinity data is possible.

### Epigenetic Mechanisms of T2DM Genetic Risk Factors and Environmental Factors in Arab Population

As mentioned earlier, the post-oil era witnessed in Arab population a rapid shift in the eating and physical activity habits. Environmental and lifestyle factors (including diet, obesity, physical activity, tobacco smoking and environmental pollutants) can influence epigenetic mechanisms, such as DNA methylation, histone acetylation, and microRNA expression; these modifications can result in altered gene expression with effects on regulation of specific genes. Epigenome-wide association studies (EWASs) that examine the role of epigenetic modifications in the etiology and progression of metabolic disorders (108–112) and diabetes (113–116) have recently emerged. Most of such EWASs with T2D and obesity are focused on Caucasian populations; however, a study emerged recently on Arab population from Qatar (117), which identified one novel CpG association at *DQX1* at genome-wide significance for T2D and replicated eight previously reported associations involving *TXNIP* for T2D and *SOCS3, SREBF1, SBNO2, CPT1A, PRR5L, LY6G6E*, and an intergenic region on chromosome 17 for BMI *albeit* at suggestive *p*-values.

## An Integrative Approach as Direction of Future T2DM Genetics Research in Arab Populations

**Figure 2:**
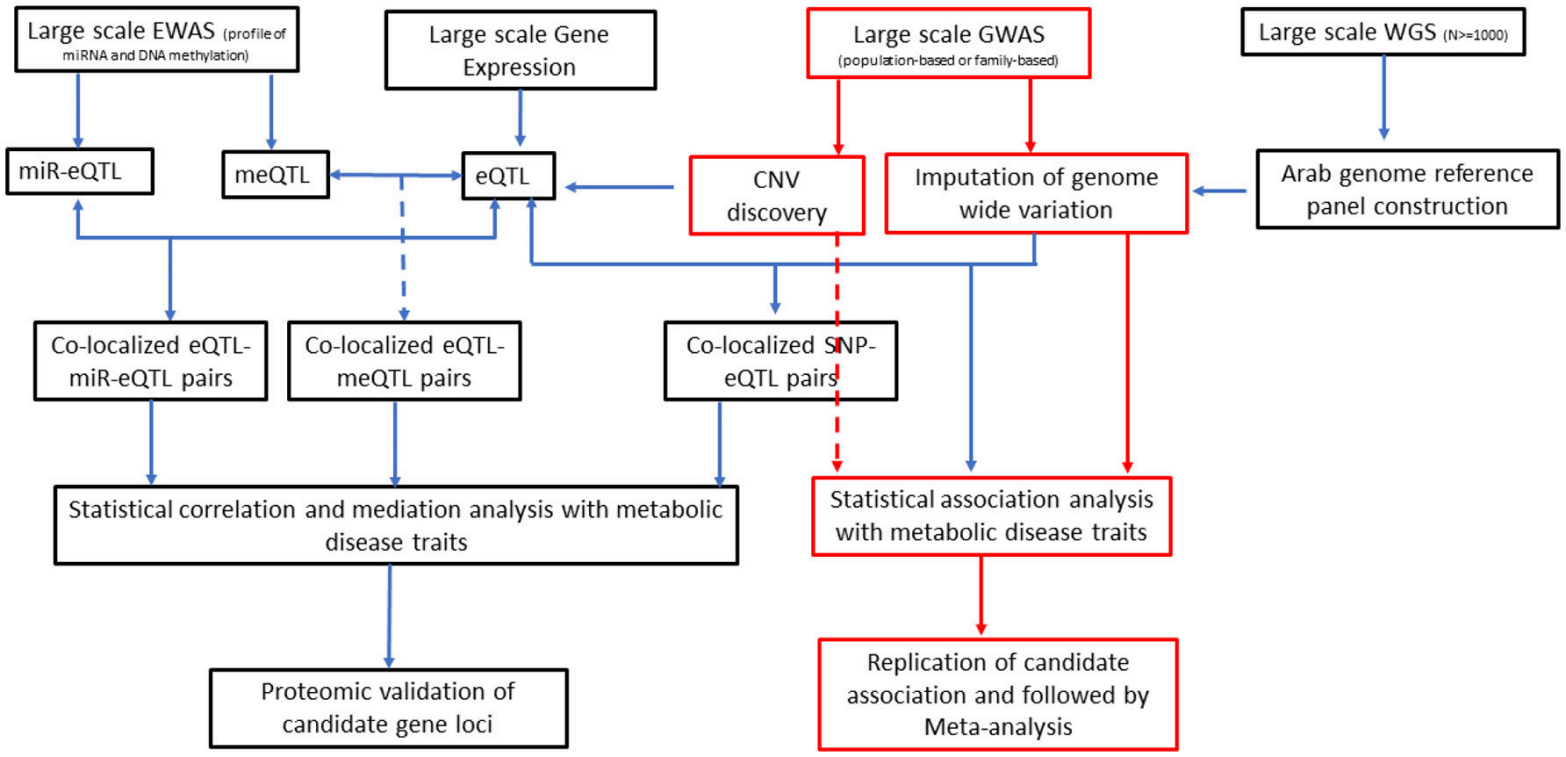
An integrative approach as direction of future T2DM genetics research in Arab populations. EWAS, epigenome-wide association studies; GWAS, genome-wide association studies; WGS, whole-genome sequencing; miR-Eqtl, microRNA expression quantitative trait loci; meQTL, methylation quantitative trait loci; eQTL, expression quantitative trait loci; CNV, copy number variation; SNP, single nucleotide polymorphism.

It has increasingly become evident that epigenetics, genetics, and environment are likely to interact with one another to define an individual's risk of diabetes and obesity (118). Integration of data on expression quantitative trait loci (eQTL), which represent regulatory loci, with genetic variants identified from GWA studies can give new insights into identification of causal genes for T2DM (119, 120). The ability of epigenetic modifications and expression of miRNA (and largely the non-coding RNAs) to manipulate gene expression has enabled incorporation of such data in research on pathogenesis of T2DM (121–123). Consideration of expression data and epigenome data along with large-scale GWA data on genotyped and imputed SNPs and copy number variations in association studies for T2DM is depicted as future directions for diabetes research (Figure 2).

## Conclusion

The failure to convincingly replicate a large number of Euro-centric risk variants for T2DM in Arab populations may have resulted from several aspects, including study design and strength, low prevalence of causative Euro-centric risk variants in the Arab population, or from the gene–environment interactions that masked the effect of the Euro-centric risk variants. However, epidemiological studies have illustrated the deficit of global risk assessment tools fitted to the Arab population (124). The performance of global genetic risk assessment tools (based on Euro-centric markers) in other populations is also questionable (125). The discrepancy of marker relevance in the applicability of Euro-centric genetic risk variants to Arab population could be resolved by performing large-scale genome-wide surveys (a combination of GWAS, exome, and genome sequencing and imputation) of the Arab population with diabetes. Detailed functional assessments of loci identified in the Arab population to interact with Euro-centric risk loci as part of common gene networks or physiologic processes should also be performed.

## Author Contributions

TT and FA-M conceptualized the study design. TT and PH developed the manuscript. All the authors participated in discussions. JA, MA-F, JT, and FA-M critically reviewed the manuscript.

### Conflict of Interest Statement

The authors declare that the research was conducted in the absence of any commercial or financial relationships that could be construed as a potential conflict of interest.
