# Supplementary material for: A Perception on Genome-Wide Genetic Analysis of Metabolic Traits in Arab Populations
Source: Front Endocrinol (Lausanne). 2019 Jan 28;10:8. doi: 10.3389/fendo.2019.00008 (PMC6362414; doi:10.3389/fendo.2019.00008)
Supplement: Supplementary file 1 [file Data_Sheet_1.PDF]

# A perception on genome-wide genetic analysis of metabolic traits in Arab populations

**Table S1. Impact of the replicated T2DM risk loci in Arab population on T2DM progression.**

| **Risk loci** | Literature annotation on the involvement of the risk loci in T2DM risk |
|---|---|
| **I. Conferring T2D risk through impaired *β*-cell function** | |
| *KCNJ11* | *KCNJ11* confers impaired pancreatic β-cell function, which seems to be the case for risk alleles in the *CDKAL1*, *SLC30A8*, *HHEX/IDE*, *CDKN2A/2B*, *IGF2BP2*, *TCF7L2*, and *KCNJ11* loci and thus affects insulin secretion. Further, both the ABCC8 and KCNJ11 genes, which encode the subunits sulfonylurea receptor 1 (SUR1) and inwardly rectifying potassium channel (Kir6.2) of the beta-cell ATP-sensitive potassium (K(ATP)) channel, control insulin secretion [1]. |
| *TCF7L2* | *TCF7L2* participates in Wnt signaling pathway and function, secretion and responsiveness of β-cells leading to impaired beta cell function [2]. |
| *WFS1* | *WFS1* is critical for survival and function of insulin-producing pancreatic β cells [3]. |
| *IGF2BP2* | *IGF2BP2* is a significant signaling molecule for growth and insulin and has been found to have effects on pancreatic development in animal models [4]. Moreover, it is found to be associated with decreased insulin secretion [5]. |
| *CDKAL1* | Variants of *CDKAL1* and *IGF2BP2* attenuate the first phase of glucose-stimulated insulin secretion [6]. *CDKAL1* disease-associated SNPs correlate with impaired insulin secretion, suggesting that β-cells in pancreatic islets may be disproportionately affected [6]. |
| *SLC30A8* | *SLC30A8* is expressed exclusively in insulin-producing β-cells (particularly in the islets of Langerhans). It encodes the largely endocrine pancreas-restricted zinc transporter *ZnT8* and the secretory granule-resident [7] and the encoded protein is involved in the storage and secretion of insulin. |

| | |
|---|---|
| *KCNQ1* | *KCNQ1 a*ffects the second-phase insulin secretion [8]. |
| *JAZF1* | Risk variants in or near *JAZF1*, *CDC123*/*CAMK1D*, and *TSPAN8* related to impairment of pancreatic β-cell function [9]. |
| ~~*HNF1,*~~ *HNF1A, HNF1B, HNF4A* | *HNF1A* is a transcription factor important for pancreatic development and beta cell differentiation and function [10].<br><br>*HNF1B* is known to influence through impaired *β*-cell function. Changes in these genes cause diabetes by reducing the amount of insulin that is produced by the pancreas. *HNF1B* participates in the regulatory networks governing pancreas development [11].<br><br>Two groups genetically engineered mice to lack *HNF4α* in β-cells ($HNF4\alpha^{loxP/loxP}$;Ins-Cre) and found that such animals exhibit normal islet architecture but defective glucose-stimulated insulin secretion in β-cells[12; 13]. |
| *GCK* | *GCK* is a key regulatory enzyme in the pancreatic β-cell, and it plays a crucial role in determining the threshold for glucose-stimulated insulin secretion [14].<br><br>All these 4 genes (*HNF1A*, *HNF1B*, *HNF4A and GCK*) are associated with both MODY and T2DM. |
| *CDKN2A/B* | *CDKN2A/B* occurs in pancreas and plays a role in the regulation of beta cell mass, proliferation and insulin secretory function [15]. |
| ***ALX4*** | *ALX4* is potentially involved in β-cell development or function [16]. |
| *BCL11A* | *BCL11A* regulates human beta cell function [17]. |
| *HMG20A* | Altered *HMG20A* expression regulates metabolism-secretion coupling genes as well as functional maturity of beta cells [18]. |
| *TP53INP1* | *TP53INP1* regulates the *TCF7L2-p53-p53INP1* pathway in such a way as to induce apoptosis and the survival of pancreatic beta cells is associated with the level of expression of *TP53INP1* [19].<br><br>Epigenomic annotations data has highlighted three putative T2D genes (*CAMK1D*, *TP53INP1* and *ATP5G1*) with plausible regulatory mechanisms [20]. |
| **II. Conferring T2D risk through impact on insulin action** | |

| | |
|---|---|
| *PPARγ* | *PPARγ* impacts through affecting peripheral insulin sensitivity [20]. |
| *ADAMTS9* | *ADAMTS9* is related to β Cell Function [21]. The impact is mediated through decreased insulin sensitivity of peripheral tissues [22]. |
| *DUSP9* | *DUSP9* encodes a member of MKP4 which plays pivotal role in regulating insulin action [23; 24]. |
| **III. Conferring T2D risk through insulin action - by primary effects on BMI, obesity and adiposity** | |
| *MC4R*, | *MC4R* is an obesity related gene and modulates insulin signaling via effects on c-Jun N-terminal kinase (*JNK*) [25] |
| *FTO* | *FTO* is an obesity-related gene and demonstrates a small but detectable influence on T2D risk through insulin action [26]. |
| *GNPDA2* | *GNPDA2* impact on type 2 diabetes risk is mediated through obesity and regulation of adiposity [27; 28]. |
| ***TFAP2B*** | *TFAP2B* variants affect the transcriptional activity of the gene in differentiated adipocytes and confer susceptibility to type 2 diabetes through the regulation of adipocytokine gene expression, such as *TNF-α*. *TNF-α* produced in adipose cells has been reported to be implicated in systemic insulin resistance [29]. |
| | |
| ***COL8A1*** | Expression of ***COL8A1*** is upregulated in diabetic nephropathy [30]; an increase in renal expression initiates other pathophysiological processes (e.g. proliferation of renal fibroblasts) involved in diabetic nephropathy. |

**Table S2. Novel Risk loci identified in our previous studies on Arab individuals from Kuwait and their biological annotation as collected from literature.**

| Metabolic traits | Gene loci/variant; Biological annotation from literature for the identified gene loci | Model | P-value; Beta value for association | OMIM annotation for the gene (where available) |
|---|---|---|---|---|
| I. **Obesity traits [31]** | | | | |
| Waist Circumference (WC) | *TCN2*/rs9606756<br><br>Interactions between the *TCN2* variant and obesity-related plasma biomarkers were seen. Further, genes harboring markers in LD with the *TCN2* marker mapped onto an interaction network (with TP53 as central element) of established obesity/diabetes-related protein components [31]. | Additive | 1.46E-07 (9.46E-08 upon correction for medication); 4.815 | Transcobalamin II deficiency (AR) (PMIM: 275350) |
| **II. Blood pressure traits [32]** | | | | |
| Systolic Blood Pressure (SBP) | *MC3R*/rs3827103 [Val81Ile]<br><br>In individuals who harbor this and LD variant, the plasma leptin levels are positively correlated with SBP and that the expression of *MC3R* is downregulated. An increase in leptin levels is known to increase sympathetic nerve activity | Additive | Sequencing the genes. 0.01; 4.9 | Mycobacterium tuberculosis, protection against; (PMIM: 607948)<br><br>Obesity, severe, susceptibility to, BMIQ9; (PMIM: 602025) |

| | | | | |
|---|---|---|---|---|
| | that, in turn, increases blood pressure [32]. | | | |
| **III. Metabolic traits [33]** | | | | |
| Glycated hemoglobin (HbA1c) | *ZNF106* (W>R)/rs12440118<br><br>*ZNF106* is a product of *SIRM* gene which has been described as a novel insulin–regulated SH3 binding protein that associates with *Grb2* and *FYN*. It could be implicated in human insulin receptor signaling pathway [33]. | Recessive | 7.07E-08; 2.006 | |
| Fasting Plasma Glucose (FPG) | *OTX2-AS1*/ rs7144734<br><br>A study on *OTX2* knockout in mice confirms that *OTX2* is indispensable for *GnRH* expression. The *GnRH* expression is found to be downregulated among T2DM men [33]**.** | Recessive | 2.82E-07; 1.465 | OTX2 with Microphthalmia, syndromic 5 (AD) (PMIM: 610125); Pituitary hormone deficiency, combined, 6 (AD) (PMIM: 613986); Retinal dystrophy, early-onset, with or without pituitary dysfunction (AD) (PMIM: 610125).<br><br>AS1 with susceptibility to |

| | | | | |
|---|---|---|---|---|
| | | | | Asthma (PMIM: 607277).<br><br>OTX2 is known to be involved in regulating gonadotrophin releasing hormone (*GnRH*) in hypogonadism. |
| Triglyceride (TG) | *PLGRKT*/rs17501809<br><br>The Plasminogen receptor with a C-terminal lysine is a transmembrane protein, known to regulate catecholamine release. Catecholamine is known to be involved in the regulation of lipoproteins including triglyceride metabolism [33]. | Recessive | 1.04E-07; 1.807 | |
| Triglyceride (TG) | *LOC105376072*/ rs11143005<br><br>We find that this genotype upregulates the downstream *PGM5* gene which is known to be involved in glucose metabolism [33]. | Recessive | 4.03E-07; 0.419 | |
| Triglyceride (TG) | *IGF1*/rs10860880<br><br>*IGF1* levels are reported to be correlated with levels of triglycerides in both human and mice. *IGF1* deficiency has been | Recessive | 2.07E-07; 1.596 | Growth retardation with deafness and mental retardation due to IGF1 |

| | | | | |
|---|---|---|---|---|
| | reported to be involved in the development of metabolic syndrome in a British cohort [33]. | | | deficiency (AR) (PMIM: 608747).<br><br>Decreased IGF-1 secretion occurs in the majority of the thalassemic patients particularly those with growth and pubertal delay [34]. Thalassemia is recessively inherited. |
| Triglyceride (TG) | [*THSD4,NR2E3*]/ rs900543<br><br>It is reported that *NR1D1*, an *NR2E3*-interacting protein, is known to regulate human *ApoC3* gene promoter, a gene that plays an important role in plasma triglyceride and remnant lipoprotein metabolism [33]. | Recessive | 1.27E-07; 1.625 | **NR2E3 with Enhanced S-cone syndrome (AR) (PMIM: 268100); Retinitis pigmentosa 37 (AD,AR) (PMIM: 611131)** |
| **IV. Lipid traits [35]** | | | | |
| Triglyceride/Fasting Plasma Glucose/Glycated hemoglobin (TG/FPG/HbA1c) | *RPS6KA1*/rs1002487<br><br>Our study points out that the TG levels have positive correlations with the insulin resistance linked trait of FPG; further that TG is associated with | Recessive | 7.17E-11; 6.517 /<br><br>1.64E-08; 8.315 | |

| | | | | |
|---|---|---|---|---|
| | higher odds of diabetes. The RSK1 protein (from *RPS6KA1*) is an important regulator of insulin signaling and glucose metabolism in the MAPK/ERK pathway. This protein can selectively phosphorylate insulin receptor substrate 1 (IRS1) and thereby prevent insulin resistance. RSK1-deficient mice remain sensitive to insulin due to the loss of the negative feedback mechanism for insulin resistance. Thus, RSK1 has the potential to be involved in insulin resistance [35]. | | | |
| Triglyceride (TG) | *LAD1*/rs11805972 | Recessive | 2.16E-17; 8.485 | Leukocyte adhesion deficiency (AR) (PMIM: 116920) |
| Triglyceride (TG) | *OR5V1*/rs7761746<br><br>A gene-based association study has implicated *OR5V1* with type 1 diabetes [36]. | Recessive | 1.31E-09; 6.006 | |
| Triglyceride (TG) | [*CTTNBP2, LSM8*]/ rs39745 | Recessive | 1.57E-08; 5.643 | |
| Triglyceride (TG) | *PGAP3*/rs2934952 | Recessive | 1.16E-09; 6.086 | Hyperphosphatasia with mental retardation |

| | | | | |
|---|---|---|---|---|
| | Association of the *PGAP3* gene loci with lipid traits were seen reported in previous GWA studies on global populations. | | | syndrome 4 (AR) (PMIM: 615716) |
| Triglyceride (TG) | [*RP11-191L9, CERK*]/ rs9626773<br><br>Ceramides play active role in glucose homeostasis, insulin signaling and, ultimately, the diabetes phenotype; ceramides in conjunction with diacylglycerols mediate high TG and insulin resistance | Recessive | 7.47E-15/7.776 | |
| Triglyceride (TG) | *ST6GALNAC5*/rs10873925<br><br>Genetic analysis of Iranian subjects demonstrated that mutations in *ST6GALNAC5* act as risk factors for coronary artery disease; epidemiological and genetic evidences exist to support the notion that raised triglyceride is an additional cause and an independent risk factor for cardiovascular disease and all-cause mortality; this notion also holds in Arab populations - serum concentrations of TG were significantly higher in the CHD+ compared to the | Recessive | 4.11E-08/0.633 | |

| | | | | |
|---|---|---|---|---|
| | CHD- group from Saudi Arabian patients. | | | |
| Triglyceride (TG) | *SPP2_ARL4C*/rs4663379<br><br>The SPP2 protein belongs to the cystatin superfamily, members of which are directly involved in diabetes and metabolic disorders. *ARLC4* has been widely implicated in the cholesterol secretion pathway and intracellular vesicular transport; it was also identified as a target of liver X receptor, which tends to play an influential role in lipid homeostasis, further indicating the significance of ARLC4 in cardiovascular diseases. | Recessive | 8.38E-09/1.841 | |
| Triglyceride (TG) | *NPY1R*/rs10033119<br><br>Involvement of *NPY1R* in metabolic disorders, including obesity, prediabetes, and prehypertension, has been established in several studies. | Recessive | 8.79E-09/2.698 | |
| Triglyceride (TG) | *LINC00911_FLRT2*/rs17709449<br><br>*FLRT2* is upregulated during adipogenesis in preadipocytes in the setting of type 2 diabetes mellitus [37]. | Recessive | 5.12E-08/1.173 | |

| | | | | |
|---|---|---|---|---|
| Triglyceride (TG) | *CDK12-NEUROD2*/ rs11654954<br><br>Polymorphisms in the Neuronal differentiation 2 (*NEUROD2)* gene have been shown to affect the onset pattern of type 1 diabetes in Japanese [38]. | Recessive | 2.18E-08, 0.9881 | |
| Triglyceride (TG) | *STARD3*/rs9972882<br><br>*STARD3* is a member of a subfamily of lipid trafficking proteins. Association of the *STARD3* gene loci with lipid traits were seen reported in previous GWA studies on global populations. | Recessive | 1.81E-08, 0.7284 | |
| | | | | |
| **V. Established markers (at genome-wide significance in GWAS Catalog) appearing in Kuwaiti data set at nominal p-values for association.** | | | | |
| Triglyceride (TG) | rs9326246/*BUD13* | Additive | 5.19E-06; 0.24 (KWT)<br><br>≤1.27E-229; 0.22 (European population [39]) | |
| **High-Density Lipoprotein (HDL)** | **rs3764261/*CETP*** | Additive | 1.10E-05 (KWT) – reached 4.64E-08 | |

| | | | | |
|---|---|---|---|---|
| | | | under joint analysis.<br><br>1E-769 (European, East Asian, South Asian and African ancestry [39]). | |
| **High-Density Lipoprotein (HDL)** | **rs1864163**/*CETP* | Additive | 4.64E-06 (KWT) – reached 1.15E-08 under joint analysis.<br><br>7E-39 (FUSION, SardINIA, Diabetes Gentics Initiative studies; also, in East Asian, and European populations [40; 41]. | |
| **High-Density Lipoprotein (HDL)** | **rs1800775**/CETP | Additive | 4.99E-06 (KWT) – reached 5.51E-08 under joint analysis.<br><br>4E-93 (Europeans and Filipinos [42]) | |

| | | | | |
|---|---|---|---|---|
| | | | | |
| Triglyceride (TG) | **rs9972882**/STARD3 | Additive | 4.07E-07 (KWT)<br><br>An LD marker rs1877031/ STARD3 is an established marker in East Asians for the related trait of HDL at 1E-21 (East Asians [40]). | |
| **VI. Established markers (at suggestive p-values in GWAS Catalog) appearing in Kuwaiti data set also at nominal p-values for association.** | | | | |
| Triglyceride (TG) | rs900543/[*THSD4*,*NR2E3*] | Recessive | 2.26E-07; 1.625 (KWT).<br><br>9.40E-05; 0.036 (Europeans [43]) Fasting insulin. | |
| Triglyceride (TG) | rs11143005/*LOC105376072* | Recessive | 3.218E-07; 0.420 (KWT).<br><br>4.47E-05; 0.11 (Europeans [44]) 2 hour fasting glucose | |

| | | | | |
|---|---|---|---|---|
| Triglyceride (TG) | rs17569297/[*LOC105369738, LOC105369739*] | Recessive | 6.963E-06; 0.773 (KWT)<br><br>1.51E-06; NA (Europeans [45]) HDL | |
| **Total Cholesterol (TC)** | rs10935794/[*RPL32P9, LINC01213*] | Additive | 3.65E-06; 0.2037 (KWT)<br><br>9.80E-05; NA (Europeans [45]) Serum ratio of Arabinosefructose | |

## References

[1] A.L. Gloyn, M.N. Weedon, K.R. Owen, M.J. Turner, B.A. Knight, G. Hitman, M. Walker, J.C. Levy, M. Sampson, S. Halford, M.I. McCarthy, A.T. Hattersley, and T.M. Frayling, Large-scale association studies of variants in genes encoding the pancreatic beta-cell KATP channel subunits Kir6.2 (KCNJ11) and SUR1 (ABCC8) confirm that the KCNJ11 E23K variant is associated with type 2 diabetes. Diabetes 52 (2003) 568-72.

[2] V. Lyssenko, R. Lupi, P. Marchetti, S. Del Guerra, M. Orho-Melander, P. Almgren, M. Sjogren, C. Ling, K.F. Eriksson, A.L. Lethagen, R. Mancarella, G. Berglund, T. Tuomi, P. Nilsson, S. Del Prato, and L. Groop, Mechanisms by which common variants in the TCF7L2 gene increase risk of type 2 diabetes. J Clin Invest 117 (2007) 2155-63.

[3] M.S. Sandhu, M.N. Weedon, K.A. Fawcett, J. Wasson, S.L. Debenham, A. Daly, H. Lango, T.M. Frayling, R.J. Neumann, R. Sherva, I. Blech, P.D. Pharoah, C.N. Palmer, C. Kimber, R. Tavendale, A.D. Morris, M.I. McCarthy, M. Walker, G. Hitman, B. Glaser, M.A. Permutt, A.T. Hattersley, N.J. Wareham, and I. Barroso, Common variants in WFS1 confer risk of type 2 diabetes. Nat Genet 39 (2007) 951-3.

[4] P. Rao, H. Wang, H. Fang, Q. Gao, J. Zhang, M. Song, Y. Zhou, Y. Wang, and W. Wang, Association between IGF2BP2 Polymorphisms and Type 2 Diabetes Mellitus: A Case-Control Study and Meta-Analysis. International journal of environmental research and public health 13 (2016).

[5] M.J. Groenewoud, J.M. Dekker, A. Fritsche, E. Reiling, G. Nijpels, R.J. Heine, J.A. Maassen, F. Machicao, S.A. Schafer, H.U. Haring, L.M. t Hart, and T.W. van Haeften, Variants of CDKAL1 and IGF2BP2 affect first-phase insulin secretion during hyperglycaemic clamps. Diabetologia 51 (2008) 1659-63.

[6] C.J. Palmer, R.J. Bruckner, J.A. Paulo, L. Kazak, J.Z. Long, A.I. Mina, Z. Deng, K.B. LeClair, J.A. Hall, S. Hong, P.H. Zushin, K.L. Smith, S.P. Gygi, S. Hagen, D.E. Cohen, and A.S. Banks, Cdkal1, a type 2 diabetes susceptibility gene, regulates mitochondrial function in adipose tissue. Molecular metabolism 6 (2017) 1212-1225.

[7] G.A. Rutter, and F. Chimienti, SLC30A8 mutations in type 2 diabetes. Diabetologia 58 (2015) 31-6.

[8] J.V. van Vliet-Ostaptchouk, T.W. van Haeften, G.W. Landman, E. Reiling, N. Kleefstra, H.J. Bilo, O.H. Klungel, A. de Boer, C.C. van Diemen, C. Wijmenga, H.M. Boezen, J.M. Dekker, E. van 't Riet, G. Nijpels, L.M. Welschen, H. Zavrelova, E.J. Bruin, C.C. Elbers, F. Bauer, N.C. Onland-Moret, Y.T. van der Schouw, D.E. Grobbee, A.M. Spijkerman, A.D. van der, A.M. Simonis-Bik, E.M. Eekhoff, M. Diamant, M.H. Kramer, D.I. Boomsma, E.J. de Geus, G. Willemsen, P.E. Slagboom, M.H. Hofker, and L.M. t Hart, Common variants in the type 2 diabetes KCNQ1 gene are associated with impairments in insulin secretion during hyperglycaemic glucose clamp. PLoS One 7 (2012) e32148.

[9] N. Grarup, G. Andersen, N.T. Krarup, A. Albrechtsen, O. Schmitz, T. Jorgensen, K. Borch-Johnsen, T. Hansen, and O. Pedersen, Association testing of novel type 2 diabetes risk alleles in the JAZF1, CDC123/CAMK1D, TSPAN8, THADA, ADAMTS9, and NOTCH2 loci with insulin release, insulin sensitivity, and obesity in a population-based sample of 4,516 glucose-tolerant middle-aged Danes. Diabetes 57 (2008) 2534-40.

[10] D.T. Odom, N. Zizlsperger, D.B. Gordon, G.W. Bell, N.J. Rinaldi, H.L. Murray, T.L. Volkert, J. Schreiber, P.A. Rolfe, D.K. Gifford, E. Fraenkel, G.I. Bell, and R.A. Young, Control of pancreas and liver gene expression by HNF transcription factors. Science 303 (2004) 1378-81.

[11] M.G. De Vas, J.L. Kopp, C. Heliot, M. Sander, S. Cereghini, and C. Haumaitre, Hnf1b controls pancreas morphogenesis and the generation of Ngn3+ endocrine progenitors. Development 142 (2015) 871-82.

[12] R.K. Gupta, M.Z. Vatamaniuk, C.S. Lee, R.C. Flaschen, J.T. Fulmer, F.M. Matschinsky, S.A. Duncan, and K.H. Kaestner, The MODY1 gene HNF-4alpha regulates selected genes involved in insulin secretion. J Clin Invest 115 (2005) 1006-15.

[13] A. Miura, K. Yamagata, M. Kakei, H. Hatakeyama, N. Takahashi, K. Fukui, T. Nammo, K. Yoneda, Y. Inoue, F.M. Sladek, M.A. Magnuson, H. Kasai, J. Miyagawa, F.J. Gonzalez, and I. Shimomura, Hepatocyte nuclear factor-4alpha is essential for glucose-stimulated insulin secretion by pancreatic beta-cells. J Biol Chem 281 (2006) 5246-57.

[14] D. Fu, X. Cong, Y. Ma, H. Cai, M. Cai, D. Li, M. Lv, X. Yuan, Y. Huang, and Z. Lv, Genetic polymorphism of glucokinase on the risk of type 2 diabetes and impaired glucose regulation: evidence based on 298,468 subjects. PLoS One 8 (2013) e55727.

[15] Y. Kong, R.B. Sharma, B.U. Nwosu, and L.C. Alonso, Islet biology, the CDKN2A/B locus and type 2 diabetes risk. Diabetologia 59 (2016) 1579-93.

[16] R. Sladek, G. Rocheleau, J. Rung, C. Dina, L. Shen, D. Serre, P. Boutin, D. Vincent, A. Belisle, S. Hadjadj, B. Balkau, B. Heude, G. Charpentier, T.J. Hudson, A. Montpetit, A.V. Pshezhetsky, M. Prentki, B.I. Posner, D.J. Balding, D. Meyre, C. Polychronakos, and P. Froguel, A genome-wide association study identifies novel risk loci for type 2 diabetes. Nature 445 (2007) 881-5.

[17] H. Peiris, S. Park, S. Louis, X. Gu, J.Y. Lam, O. Asplund, G.C. Ippolito, R. Bottino, L. Groop, H. Tucker, and S.K. Kim, Discovering human diabetes-risk gene function with genetics and physiological assays. Nature communications 9 (2018) 3855.

[18] J.M. Mellado-Gil, E. Fuente-Martin, P.I. Lorenzo, N. Cobo-Vuilleumier, L. Lopez-Noriega, A. Martin-Montalvo, I.G.H. Gomez, M. Ceballos-Chavez, L. Gomez-Jaramillo, A. Campos-Caro, S.Y. Romero-Zerbo, J. Rodriguez-Comas, J.M. Servitja, G. Rojo-Martinez, A. Hmadcha, B. Soria, M. Bugliani, P. Marchetti, F.J. Bermudez-Silva, J.C. Reyes, M. Aguilar-Diosdado, and B.R. Gauthier, The type 2 diabetes-associated HMG20A gene is mandatory for islet beta cell functional maturity. Cell death & disease 9 (2018) 279.

[19] Y. Zhou, E. Zhang, C. Berggreen, X. Jing, P. Osmark, S. Lang, C.M. Cilio, O. Goransson, L. Groop, E. Renstrom, and O. Hansson, Survival of pancreatic beta cells is partly controlled by a TCF7L2-p53-p53INP1-dependent pathway. Hum Mol Genet 21 (2012) 196-207.

[20] A. Xue, Y. Wu, Z. Zhu, F. Zhang, K.E. Kemper, Z. Zheng, L. Yengo, L.R. Lloyd-Jones, J. Sidorenko, Y. Wu, Q.C. e, A.F. McRae, P.M. Visscher, J. Zeng, and J. Yang, Genome-wide association analyses identify 143 risk variants and putative regulatory mechanisms for type 2 diabetes. Nature communications 9 (2018) 2941.

[21] M. Trombetta, S. Bonetti, M.L. Boselli, R. Miccoli, E. Trabetti, G. Malerba, P.F. Pignatti, E. Bonora, S. Del Prato, and R.C. Bonadonna, PPARG2 Pro12Ala and ADAMTS9 rs4607103 as "insulin resistance loci" and "insulin secretion loci" in Italian individuals. The GENFIEV study and the Verona Newly Diagnosed Type 2 Diabetes Study (VNDS) 4. Acta diabetologica 50 (2013) 401-8.

[22] T.W. Boesgaard, A.P. Gjesing, N. Grarup, J. Rutanen, P.A. Jansson, M.L. Hribal, G. Sesti, A. Fritsche, N. Stefan, H. Staiger, H. Haring, U. Smith, M. Laakso, O. Pedersen, T. Hansen, and E. Consortium, Variant near ADAMTS9 known to associate with type 2 diabetes is related to insulin resistance in offspring of type 2 diabetes patients--EUGENE2 study. PLoS One 4 (2009) e7236.

[23] H. Xu, M. Dembski, Q. Yang, D. Yang, A. Moriarty, O. Tayber, H. Chen, R. Kapeller, and L.A. Tartaglia, Dual specificity mitogen-activated protein (MAP) kinase phosphatase-4 plays a potential role in insulin resistance. J Biol Chem 278 (2003) 30187-92.

[24] B. Emanuelli, D. Eberle, R. Suzuki, and C.R. Kahn, Overexpression of the dual-specificity phosphatase MKP-4/DUSP-9 protects against stress-induced insulin resistance. Proc Natl Acad Sci U S A 105 (2008) 3545-50.

[25] B. Chai, J.Y. Li, W. Zhang, H. Wang, and M.W. Mulholland, Melanocortin-4 receptor activation inhibits c-Jun N-terminal kinase activity and promotes insulin signaling. Peptides 30 (2009) 1098-104.

[26] B. Xi, F. Takeuchi, G.R. Chandak, N. Kato, H.W. Pan, A.-T.D. Consortium, D.H. Zhou, H.Y. Pan, and J. Mi, Common polymorphism near the MC4R gene is associated with type 2 diabetes: data from a meta-analysis of 123,373 individuals. Diabetologia 55 (2012) 2660-2666.

[27] X. Kong, X. Zhang, Q. Zhao, J. He, L. Chen, Z. Zhao, Q. Li, J. Ge, G. Chen, X. Guo, J. Lu, J. Weng, W. Jia, L. Ji, J. Xiao, Z. Shan, J. Liu, H. Tian, Q. Ji, D. Zhu, Z. Zhou, G. Shan, and W. Yang, Obesity-related genomic loci are associated with type 2 diabetes in a Han Chinese population. PLoS One 9 (2014) e104486.

[28] M.C. Ng, C.H. Tam, W.Y. So, J.S. Ho, A.W. Chan, H.M. Lee, Y. Wang, V.K. Lam, J.C. Chan, and R.C. Ma, Implication of genetic variants near NEGR1, SEC16B, TMEM18, ETV5/DGKG, GNPDA2, LIN7C/BDNF, MTCH2, BCDIN3D/FAIM2, SH2B1, FTO, MC4R, and KCTD15 with obesity and type 2 diabetes in 7705 Chinese. The Journal of clinical endocrinology and metabolism 95 (2010) 2418-25.

[29] S. Tsukada, Y. Tanaka, H. Maegawa, A. Kashiwagi, R. Kawamori, and S. Maeda, Intronic polymorphisms within TFAP2B regulate transcriptional activity and affect adipocytokine gene expression in differentiated adipocytes. Molecular endocrinology 20 (2006) 1104-11.

[30] J. Gerth, C.D. Cohen, U. Hopfer, M.T. Lindenmeyer, M. Sommer, H.J. Grone, and G. Wolf, Collagen type VIII expression in human diabetic nephropathy. European journal of clinical investigation 37 (2007) 767-73.

[31] P. Hebbar, F. Alkayal, R. Nizam, M. Melhem, N. Elkum, S.E. John, M. Abufarha, O. Alsmadi, and T.A. Thanaraj, The TCN2 variant of rs9606756 [Ile23Val] acts as risk loci

for obesity-related traits and mediates by interacting with Apo-A1. Obesity (Silver Spring) 25 (2017) 1098-1108.

[32] O. Alsmadi, M. Melhem, P. Hebbar, G. Thareja, S.E. John, F. Alkayal, K. Behbehani, and T.A. Thanaraj, Leptin in association with common variants of MC3R mediates hypertension. American journal of hypertension 27 (2014) 973-81.

[33] P. Hebbar, N. Elkum, F. Alkayal, S.E. John, T.A. Thanaraj, and O. Alsmadi, Genetic risk variants for metabolic traits in Arab populations. Sci Rep 7 (2017) 40988.

[34] A.T. Soliman, V. De Sanctis, R. Elalaily, and M. Yassin, Insulin-like growth factor- I and factors affecting it in thalassemia major. Indian journal of endocrinology and metabolism 19 (2015) 245-51.

[35] P. Hebbar, R. Nizam, M. Melhem, F. Alkayal, N. Elkum, S.E. John, J. Tuomilehto, O. Alsmadi, and T.A. Thanaraj, Genome-wide association study identifies novel recessive genetic variants for high TGs in an Arab population. J Lipid Res (2018).

[36] Y.H. Qiu, F.Y. Deng, M.J. Li, and S.F. Lei, Identification of novel risk genes associated with type 1 diabetes mellitus using a genome-wide gene-based association analysis. J Diabetes Investig 5 (2014) 649-56.

[37] F.H. van Tienen, C.J. van der Kallen, P.J. Lindsey, R.J. Wanders, M.M. van Greevenbroek, and H.J. Smeets, Preadipocytes of type 2 diabetes subjects display an intrinsic gene expression profile of decreased differentiation capacity. Int J Obes (Lond) 35 (2011) 1154-64.

[38] S. Yamada, Y. Motohashi, T. Yanagawa, T. Maruyama, A. Kasuga, H. Hirose, K. Matsubara, A. Shimada, and T. Saruta, NeuroD/beta2 gene G-->A polymorphism may affect onset pattern of type 1 diabetes in Japanese. Diabetes Care 24 (2001) 1438-41.

[39] C.J. Willer, E.M. Schmidt, S. Sengupta, G.M. Peloso, S. Gustafsson, S. Kanoni, A. Ganna, J. Chen, M.L. Buchkovich, S. Mora, J.S. Beckmann, J.L. Bragg-Gresham, H.Y. Chang, A. Demirkan, H.M. Den Hertog, R. Do, L.A. Donnelly, G.B. Ehret, T. Esko, M.F. Feitosa, T. Ferreira, K. Fischer, P. Fontanillas, R.M. Fraser, D.F. Freitag, D. Gurdasani, K. Heikkila, E. Hypponen, A. Isaacs, A.U. Jackson, A. Johansson, T. Johnson, M. Kaakinen, J. Kettunen, M.E. Kleber, X. Li, J. Luan, L.P. Lyytikainen, P.K.E. Magnusson, M. Mangino, E. Mihailov, M.E. Montasser, M. Muller-Nurasyid, I.M. Nolte, J.R. O'Connell, C.D. Palmer, M. Perola, A.K. Petersen, S. Sanna, R. Saxena, S.K. Service, S. Shah, D. Shungin, C. Sidore, C. Song, R.J. Strawbridge, I. Surakka, T. Tanaka, T.M. Teslovich, G. Thorleifsson, E.G. Van den Herik, B.F. Voight, K.A. Volcik, L.L. Waite, A. Wong, Y. Wu, W. Zhang, D. Absher, G. Asiki, I. Barroso, L.F. Been, J.L. Bolton, L.L. Bonnycastle, P. Brambilla, M.S. Burnett, G. Cesana, M. Dimitriou, A.S.F. Doney, A. Doring, P. Elliott, S.E. Epstein, G. Ingi Eyjolfsson, B. Gigante, M.O. Goodarzi, H. Grallert, M.L. Gravito, C.J. Groves, G. Hallmans, A.L. Hartikainen, C. Hayward, D. Hernandez, A.A. Hicks, H. Holm, Y.J. Hung, T. Illig, M.R. Jones, P. Kaleebu, J.J.P. Kastelein, K.T. Khaw, E. Kim, et al., Discovery and refinement of loci associated with lipid levels. Nat Genet 45 (2013) 1274-1283.

[40] C.N. Spracklen, P. Chen, Y.J. Kim, X. Wang, H. Cai, S. Li, J. Long, Y. Wu, Y.X. Wang, F. Takeuchi, J.Y. Wu, K.J. Jung, C. Hu, K. Akiyama, Y. Zhang, S. Moon, T.A. Johnson, H. Li, R. Dorajoo, M. He, M.E. Cannon, T.S. Roman, E. Salfati, K.H. Lin, X. Guo, W.H.H.

Sheu, D. Absher, L.S. Adair, T.L. Assimes, T. Aung, Q. Cai, L.C. Chang, C.H. Chen, L.H. Chien, L.M. Chuang, S.C. Chuang, S. Du, Q. Fan, C.S.J. Fann, A.B. Feranil, Y. Friedlander, P. Gordon-Larsen, D. Gu, L. Gui, Z. Guo, C.K. Heng, J. Hixson, X. Hou, C.A. Hsiung, Y. Hu, M.Y. Hwang, C.M. Hwu, M. Isono, J.J. Juang, C.C. Khor, Y.K. Kim, W.P. Koh, M. Kubo, I.T. Lee, S.J. Lee, W.J. Lee, K.W. Liang, B. Lim, S.H. Lim, J. Liu, T. Nabika, W.H. Pan, H. Peng, T. Quertermous, C. Sabanayagam, K. Sandow, J. Shi, L. Sun, P.C. Tan, S.P. Tan, K.D. Taylor, Y.Y. Teo, S.A. Toh, T. Tsunoda, R.M. van Dam, A. Wang, F. Wang, J. Wang, W.B. Wei, Y.B. Xiang, J. Yao, J.M. Yuan, R. Zhang, W. Zhao, Y.I. Chen, S.S. Rich, J.I. Rotter, T.D. Wang, T. Wu, X. Lin, B.G. Han, T. Tanaka, Y.S. Cho, T. Katsuya, W. Jia, et al., Association analyses of East Asian individuals and trans-ancestry analyses with European individuals reveal new loci associated with cholesterol and triglyceride levels. Hum Mol Genet 26 (2017) 1770-1784.

[41] C.J. Willer, S. Sanna, A.U. Jackson, A. Scuteri, L.L. Bonnycastle, R. Clarke, S.C. Heath, N.J. Timpson, S.S. Najjar, H.M. Stringham, J. Strait, W.L. Duren, A. Maschio, F. Busonero, A. Mulas, G. Albai, A.J. Swift, M.A. Morken, N. Narisu, D. Bennett, S. Parish, H. Shen, P. Galan, P. Meneton, S. Hercberg, D. Zelenika, W.M. Chen, Y. Li, L.J. Scott, P.A. Scheet, J. Sundvall, R.M. Watanabe, R. Nagaraja, S. Ebrahim, D.A. Lawlor, Y. Ben-Shlomo, G. Davey-Smith, A.R. Shuldiner, R. Collins, R.N. Bergman, M. Uda, J. Tuomilehto, A. Cao, F.S. Collins, E. Lakatta, G.M. Lathrop, M. Boehnke, D. Schlessinger, K.L. Mohlke, and G.R. Abecasis, Newly identified loci that influence lipid concentrations and risk of coronary artery disease. Nat Genet 40 (2008) 161-9.

[42] P.M. Ridker, G. Pare, A.N. Parker, R.Y. Zee, J.P. Miletich, and D.I. Chasman, Polymorphism in the CETP gene region, HDL cholesterol, and risk of future myocardial infarction: Genomewide analysis among 18 245 initially healthy women from the Women's Genome Health Study. Circulation. Cardiovascular genetics 2 (2009) 26-33.

[43] J. Dupuis, C. Langenberg, I. Prokopenko, R. Saxena, N. Soranzo, A.U. Jackson, E. Wheeler, N.L. Glazer, N. Bouatia-Naji, A.L. Gloyn, C.M. Lindgren, R. Magi, A.P. Morris, J. Randall, T. Johnson, P. Elliott, D. Rybin, G. Thorleifsson, V. Steinthorsdottir, P. Henneman, H. Grallert, A. Dehghan, J.J. Hottenga, C.S. Franklin, P. Navarro, K. Song, A. Goel, J.R. Perry, J.M. Egan, T. Lajunen, N. Grarup, T. Sparso, A. Doney, B.F. Voight, H.M. Stringham, M. Li, S. Kanoni, P. Shrader, C. Cavalcanti-Proenca, M. Kumari, L. Qi, N.J. Timpson, C. Gieger, C. Zabena, G. Rocheleau, E. Ingelsson, P. An, J. O'Connell, J. Luan, A. Elliott, S.A. McCarroll, F. Payne, R.M. Roccasecca, F. Pattou, P. Sethupathy, K. Ardlie, Y. Ariyurek, B. Balkau, P. Barter, J.P. Beilby, Y. Ben-Shlomo, R. Benediktsson, A.J. Bennett, S. Bergmann, M. Bochud, E. Boerwinkle, A. Bonnefond, L.L. Bonnycastle, K. Borch-Johnsen, Y. Bottcher, E. Brunner, S.J. Bumpstead, G. Charpentier, Y.D. Chen, P. Chines, R. Clarke, L.J. Coin, M.N. Cooper, M. Cornelis, G. Crawford, L. Crisponi, I.N. Day, E.J. de Geus, J. Delplanque, C. Dina, M.R. Erdos, A.C. Fedson, A. Fischer-Rosinsky, N.G. Forouhi, C.S. Fox, R. Frants, M.G. Franzosi, P. Galan, M.O. Goodarzi, J. Graessler, C.J. Groves, S. Grundy, R. Gwilliam, U. Gyllensten, S. Hadjadj, et al., New genetic loci implicated in fasting glucose homeostasis and their impact on type 2 diabetes risk. Nat Genet 42 (2010) 105-16.

[44] R. Saxena, M.F. Hivert, C. Langenberg, T. Tanaka, J.S. Pankow, P. Vollenweider, V. Lyssenko, N. Bouatia-Naji, J. Dupuis, A.U. Jackson, W.H. Kao, M. Li, N.L. Glazer, A.K. Manning, J. Luan, H.M. Stringham, I. Prokopenko, T. Johnson, N. Grarup, T.W.

Boesgaard, C. Lecoeur, P. Shrader, J. O'Connell, E. Ingelsson, D.J. Couper, K. Rice, K. Song, C.H. Andreasen, C. Dina, A. Kottgen, O. Le Bacquer, F. Pattou, J. Taneera, V. Steinthorsdottir, D. Rybin, K. Ardlie, M. Sampson, L. Qi, M. van Hoek, M.N. Weedon, Y.S. Aulchenko, B.F. Voight, H. Grallert, B. Balkau, R.N. Bergman, S.J. Bielinski, A. Bonnefond, L.L. Bonnycastle, K. Borch-Johnsen, Y. Bottcher, E. Brunner, T.A. Buchanan, S.J. Bumpstead, C. Cavalcanti-Proenca, G. Charpentier, Y.D. Chen, P.S. Chines, F.S. Collins, M. Cornelis, J.C. G, J. Delplanque, A. Doney, J.M. Egan, M.R. Erdos, M. Firmann, N.G. Forouhi, C.S. Fox, M.O. Goodarzi, J. Graessler, A. Hingorani, B. Isomaa, T. Jorgensen, M. Kivimaki, P. Kovacs, K. Krohn, M. Kumari, T. Lauritzen, C. Levy-Marchal, V. Mayor, J.B. McAteer, D. Meyre, B.D. Mitchell, K.L. Mohlke, M.A. Morken, N. Narisu, C.N. Palmer, R. Pakyz, L. Pascoe, F. Payne, D. Pearson, W. Rathmann, A. Sandbaek, A.A. Sayer, L.J. Scott, S.J. Sharp, E. Sijbrands, A. Singleton, D.S. Siscovick, N.L. Smith, T. Sparso, et al., Genetic variation in GIPR influences the glucose and insulin responses to an oral glucose challenge. Nat Genet 42 (2010) 142-8.

[45] I.M. Heid, E. Boes, M. Muller, B. Kollerits, C. Lamina, S. Coassin, C. Gieger, A. Doring, N. Klopp, R. Frikke-Schmidt, A. Tybjaerg-Hansen, A. Brandstatter, A. Luchner, T. Meitinger, H.E. Wichmann, and F. Kronenberg, Genome-wide association analysis of high-density lipoprotein cholesterol in the population-based KORA study sheds new light on intergenic regions. Circulation. Cardiovascular genetics 1 (2008) 10-20.
